# Decreases in Dorsal Cervical Spinal Cord White Matter Tract Integrity Are Associated with Elevated Levels of Serum MicroRNA Biomarkers in NCAA Division I Collegiate Football Players

**DOI:** 10.1089/neur.2021.0036

**Published:** 2021-10-29

**Authors:** Linda Papa, Brian Johnson, Alexa E. Walter, James R. Wilkes, Barbara Knollmann-Ritschel, Manish Bhomia, Semyon M. Slobounov

**Affiliations:** ^1^ Department of Emergency Medicine, Orlando Regional Medical Center, Orlando, Florida, USA.; ^2^ Department of Neurology and Neurosurgery, McGill University, Montreal, Quebec, Canada.; ^3^ Department of Radiology, University of Texas Southwestern Medical Center, Dallas, Texas, USA.; ^4^ Department of Neurology, Perelman School of Medicine, University of Pennsylvania, Philadelphia, Pennsylvania, USA.; ^5^ Department of Kinesiology, Pennsylvania State University, University Park, Pennsylvania, USA.; ^6^ Department of Pathology, Uniformed Services University of the Health Sciences, Bethesda, Maryland, USA.

**Keywords:** biomarkers, cervical spinal cord, concussion, microRNA, MRI, sports

## Abstract

This prospective, controlled, observational cohort study assessed the performance of a novel panel of serum microRNA (miRNA) biomarkers relative to findings on cervical spinal cord magnetic resonance imaging (MRI) in collegiate football players. There were 44 participants included in the study: 30 non-athlete control subjects and 14 male collegiate football athletes participating in a Division I Football Bowl Subdivision of the National Collegiate Athletic Association. Diffuse tensor MRI and blood samples were acquired within the week before the athletic season began and within the week after the last game of the season. All miRNAs were significantly higher in athletes regardless of their fractional anisotropy (FA) values (*p* < 0.001), even those considered to be in the “normal” range of FA for white and gray matter integrity in the cervical spinal cord. miRNA biomarkers were most significantly correlated with FA of the white matter (WM) tracts of the dorsal (posterior) spinal cord; particularly, the fasciculus gracilis, fasciculus cuneatus, lateral corticospinal tract, rubrospinal tract, lateral reticulospinal tract, spinal lemniscus, and spinothalamic and -reticular tracts. Areas under the curve for miRNA biomarkers predicting lower FA of WM dorsal (posterior) cervical spinal tracts, therefore lower white matter integrity (connectivity), were miR-505* = 0.75 (0.54–0.96), miR-30d = 0.74 (0.52–0.95), and miR-92a = 0.75 (0.53–0.98). Should these findings be replicated in a larger cohort of athletes, these markers could potentially serve as measures of neuroimaging abnormalities in athletes at risk for concussion and subconcussive injuries to the cervical spinal cord.

## Introduction

Repetitive impacts involving the head and neck are an unfortunately common occurrence in athletes. In the United States, the nearly 8 million students who currently participate in high school athletics and the >480,000 who compete as National Collegiate Athletic Association (NCAA) athletes^1^ are at risk for concussive and subconcussive injuries.^2^ Numerous studies have documented that both clinically diagnosed concussive and repetitive subconcussive injuries induce similar changes in brain structure and functions.^3,4^ These changes, as measured by magnetic resonance imaging (MRI), include alterations in white matter (WM) and cerebrovascular integrity, blood flow, brain activation during working memory tasks, resting-state functional connectivity, and brain chemistry.^3,5,6^

Cerebral concussions have been shown to occur concurrently with neck injuries, such as cervical concussions and whiplash-associated disorders.^7–9^ These injuries have been documented after falls, motor vehicle collisions, and involvement in sports such as in wrestling, hockey, gymnastics, diving, and football.^7,10^ In contact sports, the cervical spine is particularly susceptible to injury because of axial loading forces to the head with the neck in flexion or extension. Our group recently showed that there are significant changes in WM integrity of the spinal cord with exposure to repetitive head acceleration events after a football season.^11^ However, relative to cerebral concussion, little is known about the immediate consequences and long-term effects of concussive and subconcussive injuries to the cervical spinal cord.^9^

Although there are a number of studies assessing biomarkers for traumatic spinal cord injury,^12,13^ studies examining the association between biofluid biomarkers and cervical spinal cord injury from repetitive head and neck trauma are lacking. A novel set of biomarkers, called microRNAs (miRNAs), are now being studied as the next generation of biomarkers for neurological diseases.^14^ miRNAs are small (19–28 nucleotides) endogenous RNA molecules that regulate protein synthesis at the post-transcriptional level. miRNAs are detectable in blood and are indicators of disease pathology in neuronal cells. miRNAs are relatively abundant in biofluids, such as cerebrospinal fluid, blood, and urine, and are relatively stable at variable pH conditions and temperatures and are resistant to enzymatic degradation. These properties afford miRNAs advantages over protein-based markers. The utility of miRNAs as diagnostic markers of concussion has recently been explored by our group and others and has shown significant promise.^15–20^

Based on the important functions of miRNA in neurons in the central nervous system and their association with measures of cerebral injury in adults and children, this prospective study examined the relationship between a panel of eight miRNA biomarkers previously reported by our group to correlate with neurocognition in athletes (miR-20a, miR-505*, miR-362-3p, miR-30d, miR-92a, miR-195, miR-9-3p, and miR-151-5p)^20^ with WM and gray matter (GM) regions in the anterior and posterior portions of C1 through C4 of the cervical spinal cord in collegiate football players during a single competitive season in a Division I NCAA Football Bowl Subdivision. The association between fractional anisotropy (FA) values from diffusion tensor imaging (DTI) of the cervical spinal cord and serum miRNA biomarker concentrations (obtained when neuroimaging was performed) were evaluated both pre- and post-season. We examined the ability of miRNA biomarkers to predict alterations in WM and GM integrity and assessed whether specific cervical spinal cord tracts were associated with elevations in miRNA biomarkers.

## Methods

### Study population

This prospective, controlled cohort study enrolled a sample of male collegiate student football athletes from Pennsylvania State University (University Park, PA) participating in a single competitive season in a Division I NCAA Football Bowl Subdivision. This study was approved by the Penn State Institutional Review Board, and informed consent was obtained from all participants before enrollment. Normal controls were obtained from a bank of serum from young healthy persons acquired from Bioreclamation Inc **(**BioIVT [formerly BioreclamationIVT], Westbury, NY).

### Study procedures

All participants completed a comprehensive pre-season interview, which included demographic information, medical and concussion history, and history of learning disabilities.

MRI of the cervical spinal cord along with blood samples were taken within the week before the athletic season began (before any pre- or in-season contact practices or competitions) and within the week after the last game of the regular season (post-season). None of the athletes were recovering from, or were diagnosed with, a concussion in the 9 months preceding the pre-season evaluation. None of the controls underwent MRI. DTI has become the preferred technique to objectively quantify WM microstructure through FA after sports-related concussion.

A blood sample of 5 mL was placed in a serum separator tube and allowed to clot at room temperature before being centrifuged. Serum was placed in bar-coded aliquot containers and stored at −70°C until transport to a central laboratory where samples were analyzed in batches. Lab personnel conducting the miRNA analysis were blinded to the clinical data. All athletic trainers, physicians, and research personnel were blinded to serum biomarker results.

### Outcome measures

The primary outcome measure was the association between miRNA biomarkers and FA of the WM and GM regions of cervical spinal cord. Secondary outcome measures included the performance of the biomarkers in enrolled athletes compared to non-athlete controls as well as the ability of miRNA biomarkers to predict alterations in WM and GM integrity. A tertiary outcome assessed whether specific cervical spinal cord tracts were associated with elevations in miRNA biomarkers.

During the season, athletes were monitored for cerebral concussion by certified athletic trainers and team physicians and diagnosed clinically within 24 h of injury. Study investigators then screened and triaged all injuries to confirm that they met the study definition and requirements. Concussion was defined as a type of traumatic brain injury caused by a bump, blow, or jolt to the head or by a hit to the body that causes the head and brain to move rapidly back and forth, resulting in a change in mental status, behavior, thinking, or neurological functioning based on a standardized definition.^21^

### microRNA analysis

#### RNA isolation

Serum samples from athletes (pre- and post-season) along with healthy controls were used to isolate total RNA. RNA was isolated using 100 μL of serum using a serum/plasma miRNA isolation kit (Qiagen, Hilden, Germany) as per the manufacturer's recommended protocol. RNA was eluted in 20 μL of DNAse RNAse free water and stored at −80°C for further use. For a quality check of RNA, a bioanalyzer assay using a small RNA assay was performed to confirm the quality of RNA.

#### Droplet digital polymerase chain reaction

For absolute quantitation of miRNAs, we used a droplet digital polymerase chain reaction (PCR) platform (Bio-Rad Laboratories, Hercules, CA). For droplet digital PCR reaction, 10 ng of total RNA was reverse transcribed using the specific miRNA TaqMan assays (ThermoFisherScientific, Waltham, MA), as per the recommended protocol, in a 15-μL total reaction volume. Next, 5 μL of reverse-transcribed product was used to set up the real-time PCR reaction using miRNA TaqMan assays. Then, 20 μL of the final real-time PCR reaction was mixed with 70 μL of droplet oil in a droplet generator (Bio-Rad Laboratories). After the droplet formation, the PCR reaction was performed as per the recommended thermal cycling conditions. The final PCR product within droplets was analyzed in a droplet reader (Bio-Rad Laboratories). Total positive and negative droplets were measured, and the concentration of the specific miRNA/μL of the PCR reaction was determined. All reactions were performed in duplicates.

### Magnetic resonance imaging analysis

MRI scans were performed on a Siemens 3T Prisma MR scanner (Siemens, Erlangen, Germany) with a 20-channel head/neck coil. Identical imaging protocols were obtained at both pre- and post-season that included the following sequences: two-dimensional (2D) sagittal T2-weighted fat-saturated turbo spin echo; three-dimensional axial T2*-weighted multi-echo gradient echo; and 2D axial diffusion–weighted imaging. Sagittal T2-weigthed images were acquired (echo time [TE] = 92 ms, repetition time [TR] = 3520 ms, resolution = 0.7 × 0.7 × 3.0 mm, slices = 15, fat saturation = spectral attenuated inversion recovery, acquisition time [TA] = 1:58) to evaluate cervical spine for any surrounding soft tissue or ligamentous injury. Axial multi-echo gradient echo images (TE = 17 ms, TR = 530 ms, resolution = 0.5 × 0.5 × 4.0 mm, slices = 20, magnetization transfer = yes, TA = 7:14) were acquired to provide superior GM/WM contrast for segmentation and registration. DTI metrics were computed from diffusion-weighted images acquired with a multi-segmented readout spin-echo echo-planar imaging sequence (TE = 63 ms, TR = 3000, resolution = 1.0 × 1.0 × 4.0 mm, slices = 20, b-values = 0 and 600 s/mm^2^, directions = 20, TA = 10:41) to assess WM integrity.

Image analysis was performed with the Spinal Cord Toolbox and consisted of: spinal cord segmentation, affine and non-linear registration to the MNI-Poly-AMU template, and warping of WM atlas to template. DTI images were motion corrected and coregistered, and FA was calculated. FA (value between 0 and 1) is a simplistic measure of the structural integrity of WM fiber density, axonal diameter, and myelination that describes the degree of anisotropy from diffusion. A value of zero means that diffusion is unrestricted in all directions (isotropic), whereas a value of 1 means that diffusion occurs only along one axis and is fully restricted along all other directions (anisotropic).

### Statistical analysis

Descriptive statistics with means and proportions were used to describe the data. For statistical analysis, biomarker levels were treated as continuous data, measured in copies/μL. Data were assessed for equality of variance and distribution. Pre- and post-season testing were treated as independent measurements and assessed using analysis of variance, independent-sample *t*-tests with variance consideration and the Mann-Whitney U test, when appropriate. Multiple comparisons were assessed using the Games-Howell test. Correlational analysis examined FA values against miRNA levels both pre- and post-season.

In addition to evaluating individual WM and GM tracts, the cervical spinal cord was divided into three segments for the purpose of analysis: 1) WM dorsal or posterior cervical spinal cord; 2) WM ventral or anterior cervical spinal cord; and 3) GM spinal cord. Results of the pre-season (*n* = 14) and post-season (*n* = 14) scans were compiled to yield a total of 28 scans.

Receiver operating characteristic (ROC) curves were created to evaluate the association between levels of miRNA and FA on cervical spine MRI. In order to calculate ROC curves, FA was dichotomized into lower values (FA, <0.72) signifying decreased WM and GM structural integrity (decreased connectivity) versus higher values (FA, ≥0.72) signifying better WM and GM structural integrity. This cutoff was selected based on a study by Chagawa and colleagues showing cervical spinal cord FA in healthy persons to be 0.68 ± 0.50 based on age.^22^ Given the younger age of the athletes, a more conservative estimate (higher FA value of 0.72) was selected as the cut-off level for analysis. Further, individual miRNA biomarkers were also dichotomized in order to conduct ROC curve analysis to assess whether FA values of different WM and GM regions were associated with elevations in miRNA biomarkers. Estimates of the areas under the curve (AUCs) were obtained with 95% confidence intervals (CIs; AUC = 0.5 indicates no discrimination, and an AUC = 1.0 indicates a perfect diagnostic test). All analyses were performed using the statistical software package, SPSS (version 27.0; IBM Corporation, Somers, NY).

## Results

There were 44 participants included in the study: 30 non-athlete control subjects and 14 male collegiate student football athletes. These athletes had both pre- and post-season cervical spinal cord MRI imaging for a total of 28 scans, as well as 28 blood samples available for miRNA analysis. Pre-season MRI and blood draws were completed within 1 week before the athletic season began (before any pre- or in-season contact practices or competitions) and the post-season MRI and blood draw within 1 week after the last game of the regular season. Mean age of the 14 athletes included in the analysis was 22 years (with a range from 20 to 23) with a mean height and weight of 74 inches and 262 pounds, respectively. Average number of years of football experience was 11. Six athletes (43%) had had either one or two previously diagnosed concussions (Table 1). One player was diagnosed with a concussion during the season. Control subjects were healthy adults with no chronic diseases and were a mean age of 31 years (with a range from 30 to 35), and 50% were female. There were no statistically significant differences in miRNAs between male and female control subjects except for miRNA-195, which showed higher levels in female controls (*p* = 0.043).

**Table 1.** Demographic Data for Athletes with Cervical Spinal Cord MRI

Characteristic	Athletes with cervical Spinal cord MRI* N* = 14 [95% CI]
Age	22 [21–22]
Range	20–23
Height (inches)	74 [73–76]
Weight (lbs)	263 [240–285]
Years playing football	11 [8–14]
Previous concussions	
0	8 (57%)
1	4 (29%)
2	2 (14%)
History of ADHD	1 (7%)
Player positions	
Offense	5 (36%)
Defense	9 (64%)

ADHD, attention-deficit hyperactivity disorder; CI, confidence interval; MRI, magnetic resonance imaging.

**FIG. 1.**
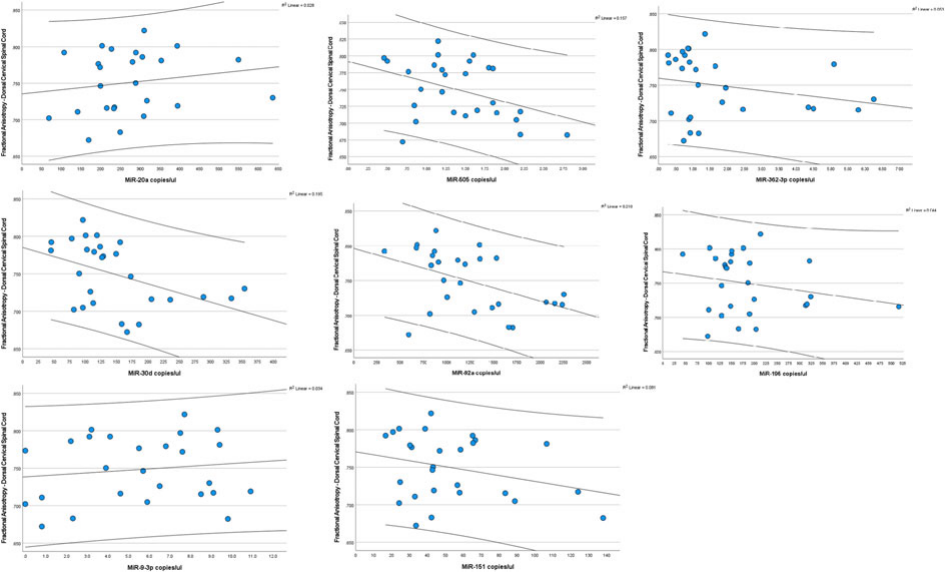
Correlation between microRNA biomarkers and fractional anisotropy of the white matter dorsal cervical spinal cord. miR-505*, miR-30d, miR-362-3p, and miR-92a were significantly and inversely correlated with FA values in the dorsal (posterior) white matter tracts of the cervical spinal cord. Lower FA values (worse connectivity) are associated with higher miRNA levels. Correlations with the other miRNAs were not significant. FA, fractional anisotropy; miR/miRNA, microRNA.

Table 2 describes correlations between the eight miRNAs and the FA from DTI of various WM tracts and GM regions assessed on cervical spinal cord, including both pre- and post-season scans. Interestingly, miR-505*, miR-30d, miR-362-3p, and miR-92a were significantly and inversely correlated with FA values in the dorsal (posterior) WM tracts of the cervical spinal cord. Hence, lower FA values (lower connectivity) were associated with higher miRNA levels. These tracts included the fasciculus gracilis, fasciculus cuneatus, lateral corticospinal tract, rubrospinal tract, lateral reticulospinal tract, and the spinal lemniscus/spinothalamic/spinoreticular tracts. These miRNAs, however, were not correlated with ventral (anterior) FA values. Correlations between the miRNAs and FA values in the dorsal (posterior) WM tracts of the cervical spinal cord are shown in Figure 1.

**Table 2.** Correlation between MicroRNAs and Fractional Anisotropy on MRI of White Matter and Gray Matter Tracts on the Cervical Spinal Cord of Football Athletes

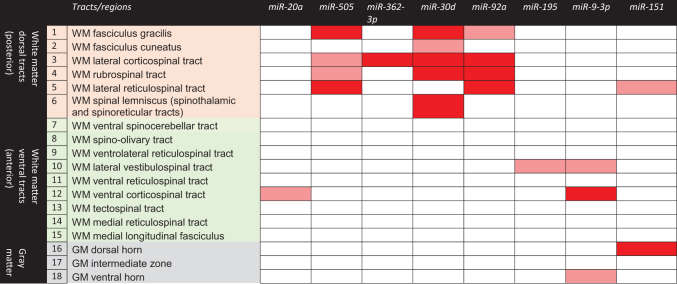

Correlation between microRNAs and fractional anisotropy on MRI of white matter tracts and gray matter regions on the cervical spinal cord of football athletes including scans from both pre- and post-season.

There was a significant inverse correlation between microRNAs and fractional anisotropy on cervical MRI (lower FA associated with higher microRNA levels). miR-505^*^, miR-30d, miR-362-3p, and miR-92a were significantly correlated (*p* < 0.05) with dorsal (posterior) white matter tracts, including the fasciculus gracilis, fasciculus cuneatus, lateral corticospinal tract, ventral spinocerebellar tract, rubrospinal tract, lateral reticulospinal tract, and spinal lemniscus/spinothalamic/spinoreticular tracts. miR-151-5p correlated with the gray matter dorsal horn, and miR-9-3p correlated with the ventral corticospinal tract.

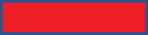
 *p* < 0.05.

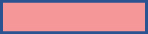
 *p* > 0.05 and *p* < 0.10.

FA, fractional anisotropy; GM, gray matter; miR, microRNA; MRI, magnetic resonance imaging; WM, white matter.

The other miRNA biomarkers (miR-20a, miR-9-3p, miR-195, and miR-151-5p) showed more-modest correlations with ventral (anterior) WM tracts and with GM regions. miR-20a and miR-9-3p correlated with the ventral corticospinal tract. miR-195 and miR-9-3p showed some correlation with the lateral vestibulospinal tract. miR-151-5p correlated with the GM dorsal horn (Table 2).

**FIG. 2.**
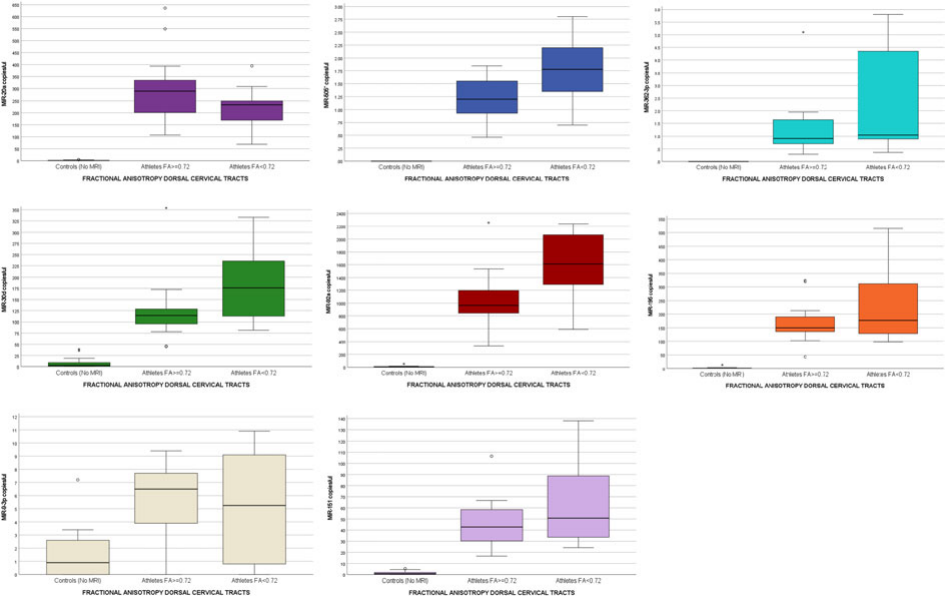
Box plot comparing of concentrations of miRNAs in controls versus athletes with lower FA values versus athletes with higher FA values in the white matter dorsal (posterior) cervical spinal tracts. There were significant differences (*p* < 0.001) in all miRNA when comparing non-athlete controls (*n* = 30) versus all athletes, including those with lower FA values (<0.72; *n* = 10 scans) and athletes with higher FA values (≥0.72; *n* = 18 scans). There was an inverse relationship between miRNAs and FA values (except for miR-20a) with elevations in miRNAs with lower FA values. When comparing low and high FA values alone (without controls and without multiple comparisons), miR-505* (*p* = 0.021), miR-30d (*p* = 0.044), and miR-92a (*p* = 0.013) were significantly higher in those with lower FA values (FA, <0.72). Box plots represent medians with interquartile ranges. FA, fractional anisotropy; miR/miRNA, microRNA.

**FIG. 3.**
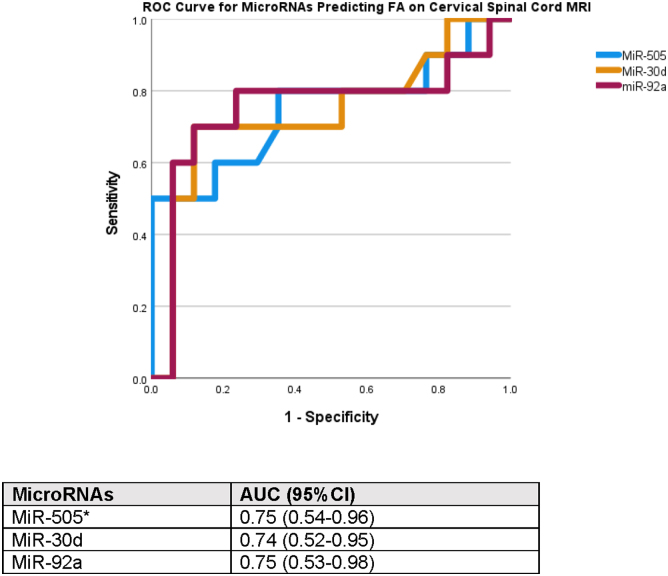
Area under the ROC curve showing the ability of microRNAs (miR-505*, miR-30d, and miR-92a) to predict decreased fractional anisotropy of the dorsal (posterior) white matter tracts of the cervical spinal cord. FA was dichotomized into lower FA values (<0.72; *n* = 10 scans) and athletes with higher FA values (≥0.72; *n* = 18 scans). Three miRNAs that had the most significant association with lower FA values in the dorsal white matter tracts included miR-505* with an AUC = 0.75 (0.54–0.96), miR-30d with an AUC = 0.74 (0.52–0.95), and miR-92a with an AUC = 0.75 (0.53–0.98). AUC, area under the curve; CI, confidence interval; FA, fractional anisotropy; miR/miRNA, microRNA; MRI, magnetic resonance imaging; ROC, receiver operating characteristic.

Concentrations of miRNA biomarkers were compared between three groups: 1) non-athlete control subjects; 2) athletes with higher FA values (FA, ≥0.72); and 3) athletes with lower FA values (FA, <0.72) in the WM dorsal (posterior) cervical spinal tracts (Fig..2). There were significant differences in miRNA concentrations between all three groups (*p* < 0.001). All miRNA biomarkers were significantly higher in athletes, compared to controls, regardless of their FA values, even those considered to be in the “normal” range of FA for dorsal WM integrity. When comparing athletes with low to high FA values alone (without controls and without multiple comparisons), biomarkers miR-505* (*p* = 0.021), miR-30d (*p* = 0.044), and miR-92a (*p* = 0.013) were significantly higher in those with lower FA values (FA, <0.72). Areas under the ROC curve for the ability of these miRNA biomarkers to predict lower FA (lower WM integrity or connectivity) in the WM dorsal (posterior) cervical spinal tracts were: AUC = 0.75 (95% CI, 0.54–0.96) for miR-505*; AUC = 0.74 (95% CI, 0.52–0.95) for miR-30d; and AUC = 0.75 (95% CI, 0.53–0.98) for miR-92a (Fig. 3).

Differences between miRNA biomarkers between non-athlete control subjects and athletes for the ventral (anterior) spinal cord and GM regions showed a similar pattern. All eight miRNA biomarkers were significantly higher in athletes regardless of their FA values (*p* < 0.001), even those considered to be in the normal range of FA for WM and GM integrity in the cervical spinal cord. This is consistent with a recent study which showed that decreases in FA is moderated by the number of previous concussions and head acceleration events >80*g* over a season in football players.^11^

**FIG. 4.**
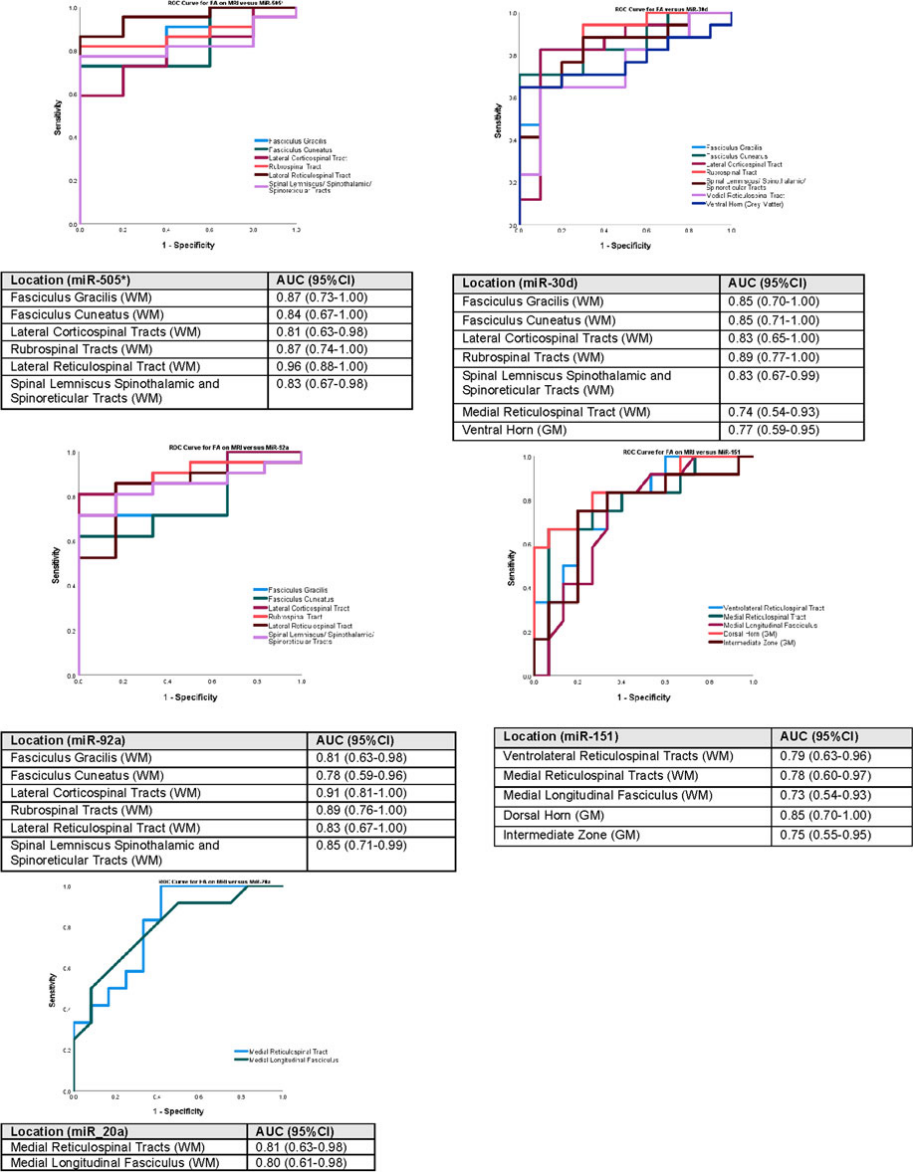
Area under the ROC curve showing how FA values of different cervical spinal cord tracts could predict threshold elevations in miRNA biomarkers. To further evaluate the relationship between decreasing FA values (lower white matter integrity) of specific cervical spinal cord tracts in football athletes and miRNA biomarkers, ROC curves were constructed to depict how the FA could predict threshold elevations in miRNA biomarkers. Each miRNA biomarker was dichotomized based on the ROC curve and compared to the FA of different cervical spinal cord tracts/regions. All AUCs presented in the figures are statistically significant (*p* < 0.05). AUC, area under the curve; CI, confidence interval; FA, fractional anisotropy; miR/miRNA, microRNA; MRI, magnetic resonance imaging; ROC, receiver operating characteristic; WM, white matter.

To further evaluate the relationship between decreasing FA values (lower WM integrity) of specific cervical spinal cord tracts in football players and miRNA biomarkers, ROC curves were constructed to depict how FA could predict threshold elevations in miRNA biomarkers. Each miRNA biomarker was dichotomized based on the ROC curve and compared to the FA of different cervical spinal cord tracts/regions (Fig. 4). Elevations in miR-505* >1.9 copies/μL were predicted by decreasing FA values with AUCs in the fasciculus gracilis of 0.87, fasciculus cuneatus 0.84, lateral corticospinal tract 0.81, rubrospinal tracts 0.87, lateral reticulospinal tract 0.96, and spinal lemniscus/spinothalamic/spinoreticular tracts 0.83. Elevations in miR-30d >150 copies/μL were predicted by decreasing FA values with AUCs in the fasciculus gracilis 0.85, fasciculus cuneatus 0.85, lateral corticospinal tract 0.83, rubrospinal tract 0.89, spinal lemniscus/spinothalamic/spinoreticular tracts 0.83, medial reticulospinal tract 0.74, and ventral horn (GM) 0.77. Elevations in miR-92a >1600 copies/μL were predicted by decreasing FA values with AUCs in the fasciculus gracilis 0.81, fasciculus cuneatus 0.78, lateral corticospinal tract 0.91, rubrospinal tract 0.89, lateral reticulospinal tract 0.83, and spinal lemniscus/spinothalamic/spinoreticular tracts 0.85.

In contrast, miR-151-5p and 20a were positively correlated with FA and therefore decreased (downregulated) with decreases in FA. Decreases in miR-151-5p <45 copies/μL were predicted by decreasing FA values with AUCs in the ventrolateral reticulospinal tract 0.79, medial reticulospinal tract 0.78, medial longitudinal fasciculus 0.73, dorsal horn (GM) 0.85, and intermediate zone (GM) 0.75. Decreases in miR-20a <250 copies/μL were predicted by decreasing FA values with AUCs in the medial reticulospinal tract 0.81 and medial longitudinal fasciculus 0.80.

## Discussion

This prospective study examined DTI of the cervical spinal cord along with levels of serum miRNA in collegiate athletes with an average of 11 years of playing experience who were asymptomatic but experienced multiple, high-intensity subconcussive impacts over the course of their playing career. This panel of eight miRNA biomarkers (miR-20a, miR-505*, miR-362-3p, miR-30d, miR-92a, miR-195, miR-9-3p, and miR-151-5p), which have previously been shown to correlate with neurocognitive function in collegiate football players,^20^ showed significant correlations with decreases in FA in the tracts of the cervical spinal cord, particularly the dorsal (posterior) WM tracts. Given that blood sampling and neuroimaging were conducted within the same period of time pre- and post-season, the correlations between these two modalities were temporally synchronized. This study is among the first to evaluate changes in the cervical spinal cord in athletes exposed to repetitive head acceleration events and correlate WM integrity with miRNA biomarkers.

These findings reflect axonal disruption and demyelination in specific WM tracts within the posterior cervical spinal cord of Division I football players and introduce miRNA biomarkers as potential markers of cervical spinal cord injury from repetitive impacts in clinically asymptomatic players. These data are an extension of our work showing pre- and post-season changes in miRNA^20^ and WM integrity.^11^

Studies of sports-related concussive and subconcussive injuries have typically emphasized cerebral or brain injury; however, recent research has shown that cervical injury may be an important area of damage from repetitive impacts.^8^ This study adds to the growing literature that asymptomatic cervical spinal cord injury occurs in athletes with a history of repetitive trauma.^11^ Further, it suggests that the posterior or dorsal WM tracts appear particularly vulnerable to injury. In contact sports, posterior cervical spinal cord injuries are often associated with axial loading forces to the head while the neck is in flexion.^23,24^ However, there is also potential for injury while the neck is in extension.^23,24^ Additionally, WM tracts of the cervical spine have been reported to be particularly vulnerable to damage from rotational forces.^23–25^ Pre-clinical models and clinical imaging research of subconcussive spinal cord injury could help elucidate this.

WM changes from pre- to post-season in Division I football players have been shown by our group to be moderated by previous concussion history and exposure to impacts despite athletes not experiencing a concussion (either cerebral of cervical).^11^ Therefore, repetitive impacts to the head and neck in contact sports have major implications on the diagnosis, management, and monitoring of subclinical cerebral and cervical spinal cord injury. Given that injury to the cervical spinal cord is often overlooked in sports-related concussion, it is even more important to monitor them objectively with tools such as DTI and biofluid biomarkers. Moreover, these miRNA biomarkers could potentially serve as surrogate measures of injury if validated correlations between DTI and miRNA can be made.

Interestingly, the miRNAs (particularly miR-505*, miR-30d, and miR92a) were most indicative of injury to the dorsal (posterior) WM tracts of the cervical spinal cord. Although there were a few miRNAs, including miR-151-5p, miR-9-3p, and miR-20a, that did show some correlation with ventral or anterior WM and GM tracts, the association was not as robust. The specific WM tracts most closely associated with elevations in miR-505*, miR-30d, and miR-92a were fasciculus gracilis, fasciculus cuneatus, lateral corticospinal tract, rubrospinal tract, lateral reticulospinal tract, and the spinal lemniscus/spinothalamic/spinoreticular tracts.

When concentrations of the miRNA biomarkers were compared between non-athlete control subjects and athletes with high and low FA values, all miRNA biomarkers were significantly higher in athletes regardless of their FA values, even those considered to be in the normal range of FA (*p* < 0.001). This suggests that these football players have higher baseline levels of miRNAs than those who do not participate in sports.^20^ Cumulative effects of repetitive subconcussive impacts are a subject of great interest in sports.^26^ Research now suggests that head impacts commonly occur during contact sports, and visible signs or symptoms of neurological dysfunction may not be evident despite those impacts having the potential for neurological injury.^5,26^ This includes studies in children ages 8–13 years who play football^6^ as well as those who play high school football.^27^

The fact that miR-505*, miR-30d, and miR-92 are associated with dorsal WM integrity is remarkable because these markers have also shown a strong correlation to neurocognitive outcome pre- and post-season in a previous study by our group.^20^ More specifically, miR-505* was strongly associated with problems of balance and miR-92a by reaction time.^20^ These findings are consistent with others who have found that athletes exposed to repetitive subconcussive head impacts are at greater risk for lowered neurocognitive functioning.^28^

These posterior tracts of the cervical spinal cord include both motor and sensory fibers that are part of a continuum that begin in the brain and work their way down the spinal cord, conveying information about movement, proprioception, touch, vibration, pain, and temperature. Whiplash-associated disorders and concussion have traditionally been treated as separate entities; however, they may have similar presenting symptoms, biomechanical mechanisms, and neurophysiological sequelae.^8,9^ Currently, guidelines for the diagnosis and management of whiplash and concussion have been developed and implemented separately, and the definitive diagnosis for either remains elusive. Perhaps future clinical and research frameworks should consider these entities as a continuum.

Repetitive impacts and concussions in contact sports are common, but they can be elusive because athletes often do not report their symptoms.^29^ Biomarkers, through a simple blood test, could provide a more objective measure of injury, potentially identifying athletes at risk for declining WM tract integrity and neurocognition^20,30–35^ and may lend important monitoring information as well.^34^ Given our previous work showing that athletes with a previous history of concussion had loss of WM integrity in the cervical spinal cord post-season,^11^ an accumulative effect of damage to the cervical spinal cord may need to be monitored. Although not studied here, medical decisions about athlete resumption or continuation of sports activities is a potential use of DTI and biomarkers.

### Limitations

Although these data are encouraging, we recognize that there are limitations to this study. Athletes were enrolled for a single season at a single site and represent a small sample of athletes. The players, however, are representative of many players in this Division of the Football Bowl Subdivision of the NCAA. In order to maximize the sample size for the analysis, we compiled the pre- and post-MRI results, yielding a total of 28 scans to look at correlations between miRNA and FA. Separate published studies by our group have already shown pre- and post-season changes in miRNA^20^ and WM.^11^

We cannot confirm that the selected miRNAs are brain- or spinal-cord specific and may be released from other organs. However, in this study, levels of selected miRNAs in healthy controls were significantly lower than in athletes. A previous study by our group comparing mild TBI to trauma control subjects have shown good discrimination between trauma controls and mild TBI for the selected miRNA markers.^16^ According to the data from a recent report based on miRNA sequencing analysis of various human organs, miR-9-3p, miR-151-5p, and miR-195 are highly enriched in the brain. Additionally, miRNAs miR-20a, miR-30d, miR-92a, miR-505*, and miR-362-3p are reported to be expressed in the human brain.^36,37^ Further study is needed to examine this.

Further limitations include a lack of access to the concussion history of the healthy controls and an inability to assess sex differences among athletes given that they were all male. There were no statistically significant differences among sexes in the control group except for miRNA-195, which showed higher levels in females. The range in ages between athletes and control subjects was slightly different (19–24 vs. 30–35, respectively). Age and sex differences need further exploration, and studies are currently underway.

## Conclusion

This study introduces a panel of miRNA biomarkers, previously shown to correlate with acute concussion and neurocognitive functioning, as potential markers of cervical spinal cord injury from repetitive impacts. miR-505*, miR-30d, and miR-92 were associated with dorsal WM tract integrity, particularly the fasciculus gracilis, fasciculus cuneatus, lateral corticospinal tract, rubrospinal tract, lateral reticulospinal tract, and the spinal lemniscus/spinothalamic/spinoreticular tracts. This study adds to the growing literature that asymptomatic cervical spinal cord injury occurs in athletes with a history of repetitive trauma. Further, it suggests that the posterior or dorsal WM tracts appear particularly vulnerable to injury. Should these findings be replicated in larger, more-diverse cohorts of athletes, these markers could potentially serve as measures of neuroimaging abnormalities in athletes at risk for concussion and subconcussive injuries to the cervical spinal cord.

## Abbreviations Used

2D: two-dimensional
AUCs: areas under the curve
CIs: confidence intervals
DTI: diffusion tensor imaging
FA: fractional anisotropy
GM: gray matter
miRNAs: microRNAs
MRI: magnetic resonance imaging
NCAA: National Collegiate Athletic Association
PCR: polymerase chain reaction
ROC: receiver operating characteristic
TA: acquisition time
TE: echo time
TR: repetition time
WM: white matter
